# A novel approach for adsorption of organic dyes from aqueous solutions using a sodium alginate/titanium dioxide nanowire doped with zirconium cryogel beads

**DOI:** 10.3389/fchem.2024.1285230

**Published:** 2024-03-13

**Authors:** Sana Azeroual, Khalid Khatib, Ahmed Belfkira, El-Houssaine Ablouh, Zouhair Hanani, Moha Taourirte, Rachid Jalal

**Affiliations:** ^1^ Laboratory of Sustainable Development and Health Research, Department of Chemistry, Faculty of Sciences and Technology, Cadi Ayyad University, Marrakesh, Morocco; ^2^ Center for Agrobiotechnology and Bioengineering, CNRST Labeled Research Unit (Centre AgroBiotech, URL-CNRST 05), Cadi Ayyad University, Marrakech, Morocco; ^3^ Advanced Materials Department, Jožef Stefan Institute, Ljubljana, Slovenia; ^4^ Jozef Stefan Institute, Ljubljana, Slovenia

**Keywords:** adsorption, biopolymer, cryogel, organic dyes, sodium alginate, zirconium-doped titanium dioxide

## Abstract

The presence of organic dyes in wastewater raises significant environmental and human health concerns, owing to their high toxicity. In light of this, a novel adsorbent material with porous cryogel architecture was developed and employed for the effective removal of organic dyes from an aqueous solution. Initially, a titanium dioxide nanowire doped with zirconium HZTO was synthesized by the hydrothermal process. Subsequently, the beads (SA/HZTO) of sodium alginate and HZTO were successfully prepared through a cross-linking process, employing Ca^2+^ ions as the crosslinking agent. Structural analysis of SA/HZTO beads was performed using FTIR, SEM, and EDX techniques. We systematically examined the impact of different conditions, including the initial dye concentration, pH, contact time, and adsorbent dosage, on the adsorption process. Batch experiments, both in signal and binary systems, were conducted to rigorously assess the dye adsorption capabilities. Kinetic modeling revealed that the adsorption process adhered to the pseudo-second-order kinetic model. Remarkably, the prepared beads exhibited impressive adsorption capacities of 26 and 29 mg/g toward methylene blue (MB) and safranin (SF), respectively. SA/HZTO beads have demonstrated excellent adsorption properties, offering a promising avenue for the development of low-cost, efficient, and reusable adsorbent to remove dyes from wastewater.

## 1 Introduction

The most crucial natural resource on which humans depend for survival is commonly acknowledged to be water resources. However, the rapid industrialization and the rapid growth of population, water crisis, and pollution are becoming urgent issues that need to be remedied. Nowadays, the demand for organic dyes is constantly increasing. These products are commonly used in many industries including textiles, leather, paints, pharmaceuticals, and tanneries (Ben Yahia and Sellaoui, 2020). Organic dyes used in the printing industries and dyeing are accompanied by their presence in wastewater harmful to human health and biota. Indeed, these coloring molecules minimized the transparency and esthetic value of water, which reduces light penetration, thus affecting photosynthesis (Li et al., 2021), and raises the chemical oxygen demand (COD) (Flores-Gómez et al., 2023), due to their extreme stability, making it difficult to break them down using light and oxidation reactions (Somma et al., 2021). Additionally, these dyes are allergenic, carcinogenic, and non-degradable (Laysandra et al., 2017). Consequently, there has been an increase in interest in the design and development of innovative technologies for the removal of dye pollutants from wastewater such as oxidation and biological treatment. These methods are expensive and complicated. Adsorption has been found to be the best removal process due to its high efficiency, wide applicability, and low cost (Sirajudheen et al., 2020). Currently, the elimination of water-soluble toxic substances by an environmentally friendly process is a challenge for scientists. We must discover new ways and the decontamination technique that are ecological, efficient, and inexpensive at the same time.

The elimination of organic dyes in aqueous solutions by adsorption has proved to be an efficient and easy-to-use means (Zhang et al., 2012). Adsorption is defined as a physical or chemical contact between a solute (adsorbate) and the surface of a solid (adsorbent). Large volumes of water can be treated with this method in an easy-to-use manner at a reasonable cost. The ability to recycle and repurpose the adsorbent material is one benefit of adsorption. A solid with convenient and easy regeneration is an excellent contender for this purpose (Somma et al., 2021). Thus, several research works have been devoted to the exploration of new adsorbents, using natural materials that are less expensive, widely available, biodegradable, and easy to regenerate (Duan et al., 2018; Chaouf et al., 2019; Ablouh et al., 2020; Achour et al., 2021; Nasrollahzadeh et al., 2021; Salim et al., 2022). Due to their inherent qualities, such as their high surface zone, high specific surface area, structural and functional bioavailability, and large porosity and geometry tolerability, a new generation of distinctive and useful cryogel materials has recently gained a lot of attention in water treatment (Gao et al., 2020). Cryogels excel as a versatile material due to their unique characteristics. These substances are composed of polymers that have undergone chemical or physical crosslinking. This inherent quality renders cryogels highly valuable in the realm of wastewater treatment (Benettayeb et al., 2022). Cryogels find extensive use in various fields, including drug delivery, water treatment, catalysis, and chemical separation processes (Mallepally et al., 2013). Natural polymeric cryogels offer a distinct advantage over synthetic polymers, due to their inherent biodegradability and biocompatibility (Aaliya et al., 2021).

Cryogel, a type of adsorbent, can prevent the technical issues associated with recovering powder adsorbents and secondary contamination. For examples, without being exhaustive: chitosan–resole–pectin aerogel (Flores-Gómez et al., 2023), magnetic gel beads based on polyanetholesulfonic acid/alginate/magnetic zeolite (Metin et al., 2020), K-carrageenan and alginate (Ammar et al., 2021), chitosan (Khnifira et al., 2021), magnetic nanoparticles of chitosan-glyoxal/ZnO/Fe₃O₄ crosslinked using a Schiff base (Reghioua et al., 2021), fibrous composite foams chitosan/sodium alginate (Zhao et al., 2020), calcium alginate-immobilized graphene oxide composites (Li et al., 2013), MXene/PEI-functionalized sodium alginate (Feng et al., 2021), and amino-functionalized sodium alginate (Wang et al., 2022). The use of alginate more than other biopolymers is due to several considerations. First of all, it is a polymer that we have mastered its extraction and use (Fertah et al., 2017; Boussetta et al., 2021a). Alginate is a naturally abundant polyanionic polysaccharide produced from brown algae (Browning et al., 2021), (Boussetta et al., 2021b). The alginate has a linear structure consisting of 1,4-L-guluronic acid and 1,4-β-D-mannuronic acid, with heteropolymeric and homopolymeric blocks, respectively (Aguilar et al., 2015). Sodium alginate can interconnect with multivalent cations, such as Ca^2+^, Fe^3+^, and Ba^2+^, forming aerogel beads with an “egg-box” structure (Kong et al., 2020). It is an excellent adsorbent with very high removal capacities for organic and cationic pollutants, due to its contents of carboxylic and hydroxyl functions (Nouri et al., 2020). The efficient adsorption capacity of cross-linked sodium alginate has been decreased, due to the interaction of carboxyl groups with the crosslinking agent (Tao et al., 2020). Additionally, enhancing the mechanical properties of alginate aerogel beads is also presently a significant challenge. Therefore, there is a growing focus on creating composites of SA with other materials to achieve a highly efficient adsorbent.

The other component of the material used in this study is doped titanium dioxide. Indeed, this pigment is known for its opacifying properties in the field of paints, its non-toxicity, its low cost, and its availability (Kanakaraju et al., 2017). Recently, the incorporation of TiO₂ in adsorbent systems has made it possible to increase their specific surfaces (Lai et al., 2020). Compared to the adsorption performance of two systems (xanthan/graphene oxide and multi-wall carbon nanotubes), the incorporation of TiO₂ made it possible to increase the adsorption efficiency by creating additional active sites (Lai et al., 2020), (Zhao et al., 2010). This research aims to examine the effectiveness of new composites based on a titanium dioxide nanowire doped with zirconium and sodium alginate. The specific objectives were to 1) prepare and characterize HZTO and SA/HZTO beads; 2) determine the adsorption kinetics and capacities of the SA/HZTO adsorbent; 3) study the effects of parameters such as the initial dye concentration, the initial pH of the solution, and the state of ionization of the species present, in light of the adsorption rates obtained; and 4) elucidate the underlying removal mechanisms. Kinetic and thermodynamic models which match the results obtained will be examined.

## 2 Experiment

### 2.1 Materials

Sodium alginate (molecular weight, 216 KDa), hydrochloric acid (HCl, 37%), sodium hydroxide, methylene blue, and ethanol were bought from Sigma-Aldrich. Calcium chloride (CaCl₂) was purchased from VWR chemicals. Safranin was purchased from Fluka Chemicals.

### 2.2 Preparation of H₂(Zr₀.₁Ti₀.₉)₃O₇ nanowires

H_2_ (Zr_0.1_Ti_0.9_)_3_O_7_ nanowires (HZTO-nw) were prepared by dispersing 5 g of Zr_0.1_Ti_0.9_O_2_ (ZTO), prepared by the method described by Hanani et al. (2018), in NaOH (10 M, 100 mL). After 1 h of stirring, the suspension was placed in 150-mL Teflon-lined stainless-steel autoclave, sealed and heated to 240°C with 48 h dwell time, and then allowed to cool. The suspension of Na_2_(Zr_0.1_Ti_0.9_)_3_O_7_ (NaZTO) was filtered and hardened in the HCl (0.2 M) aqueous solution for 10 h to transform NaZTO to HZTO. Then, HZTO nanowires were purified several times with ethanol and deionized water by centrifugation at 4,000 rpm for 10 min. The resulting HZTO nanowires were dispersed in deionized water under mechanical agitation at 60 rpm for 30 min and then freeze-dried for 48 h.

### 2.3 Preparation of SA/HZTO beads

The SA/HZTO beads were prepared in two steps. First, we prepared a dispersion and a solution of nanowires and alginate, respectively. For the nanowires, 0.1 g of HZTO was dispersed in 50 mL of distilled water with vigorous agitation for 24 h. 3 g of sodium alginate, was solubilized in 100 mL of distilled water under magnetic stirring for 24 h. In the second step, the dispersion of nanowires is poured into the alginate solution with vigorous stirring. The mixture obtained is kept under magnetic agitation for 24 h to collect a gel of SA/HZTO. The resulting dispersion was added dropwise to the 0.1 M calcium chloride solution. The SA/HZTO beads were purified with distilled water, and subsequently, it was subjected to freeze-drying for characterization.

### 2.4 Point of zero charge (pH_pzc_) determination

The pH_pzc_ value of SA and SA/HZTO was determined by the pH drift method. A measure of 50 mL of the NaCl solution (0.01 M) was added to nine vials. The initial pH of the solution in the vials was adjusted from 2 to 10 using 0.1 M NaOH or HCl to each of the solutions; then, 500 mg SA/HZTO or SA was poured into the solution and stirred for 24 h; subsequently, the final pH was measured.

### 2.5 Characterization

The crystalline composition of the HZTO sample was examined by X-ray diffraction (XRD, Rigaku SmartLab) using a step angle of 0.012° in the 2θ range from 5° to 80° and Cu-Kα radiation (*λ* ≈ 1.54059Å). The resulting morphologies of the HZTO sample were analyzed using a scanning electron microscope (FESEM, JEOL JSM-7600F) provided with an electron gun and a high-resolution transmission electron microscope (HRTEM, JEOL—ARM 200F Cold FEG) operating at 200 kV and provided with a spherical aberration (Cs) probe and image correctors with a point resolution of 0.12 nm. Energy-dispersive X-ray spectroscopy (EDS) and energy-filtered transmission electron microscopy (EFTEM) elemental mapping techniques were employed to explore the compositions of the producing nanostructures. Fourier transform infrared spectroscopy (FTIR, Jasco-6030) operated from 4,000 to 400 cm^−1^ at 4 cm^−1^ step by attenuated total reflection (ATR) was used to analyzed the functional groups of samples. The surface morphologies of the SA/HZTO bead surface and cross section were determined using a scanning electron microscope (SEM, TESCAN, Vega3), with an acceleration voltage of 20 KV.

### 2.6 Adsorption study

Typical methylene blue (MB) and safranin (SF) cationic dyes were chosen to assess the removal capacity of SA/HZTO beads. The residual concentrations of SF and MB were analyzed with a UV-vis spectrophotometer (UV3100PC) at wavelengths of 512 and 664 nm, respectively. The impact of the different parameters, influencing the adsorption phenomenon, was studied in the following ranges: pH (3–10), temperature (30°C–60°C), a dose of adsorbent (300–800 mg), and dye concentration (5–20 mg/L). The pH of the solution was regulated with 0.1 M NaOH and HCl. In each experimental run, 500 mg of the adsorbent was placed in 20 mL of a colorant aqueous solution. The organic dye removal efficiency (RE) and dye adsorption capacity (qe) by SA/HZTO were determined using Eqs 1, 2:

$$q_{e}=\left(\frac{C₀-C_{e}}{W}\right)\;V,$$ (1)

$$\text{RE}\left(\%\right)=\frac{C₀-C_{e}}{C₀}\times 100,$$ (2)

where C₀ (mg/L) and C_e_ (mg/L) are the initial and equilibrium concentrations of the dye solution, respectively; V (L) is the volume of the dye solution; and w (g) is the weight of the beads.

Competitive adsorption is determined by the affinity of the gel beads for the MB and SF dyes. For this experiment, 500 mg of SA/HZTO is added to the 20-mL sample of a mixture solution of the two dyes at different ratios: [MB (20 mg/L) + SF (5 mg/L)], [MB (20 mg/L) + SF (10 mg/L)], [MB (10 mg/L) + SF (20 mg/L)], [MB (5 mg/L) + SF (20 mg/L)], and [MB (20 mg/L) + SF (20 mg/L)]. After the adsorption phenomenon, the removal capacity of each dye is measured as described above.

For regeneration, the adsorbed SA/HZTO beads from SF and MB dye solutions were immersed for 1 h in 0.1 mol/L HCl to remove the adsorbed dyes. The recycled samples were washed by distilled water. Subsequently, the beads were used for the next adsorption experiment. This recycling procedure was conducted three times to evaluate the reusability of SA/HZTO beads.

## 3 Result and discussion

### 3.1 HZTO nanowire characteristics

**FIGURE 1:**
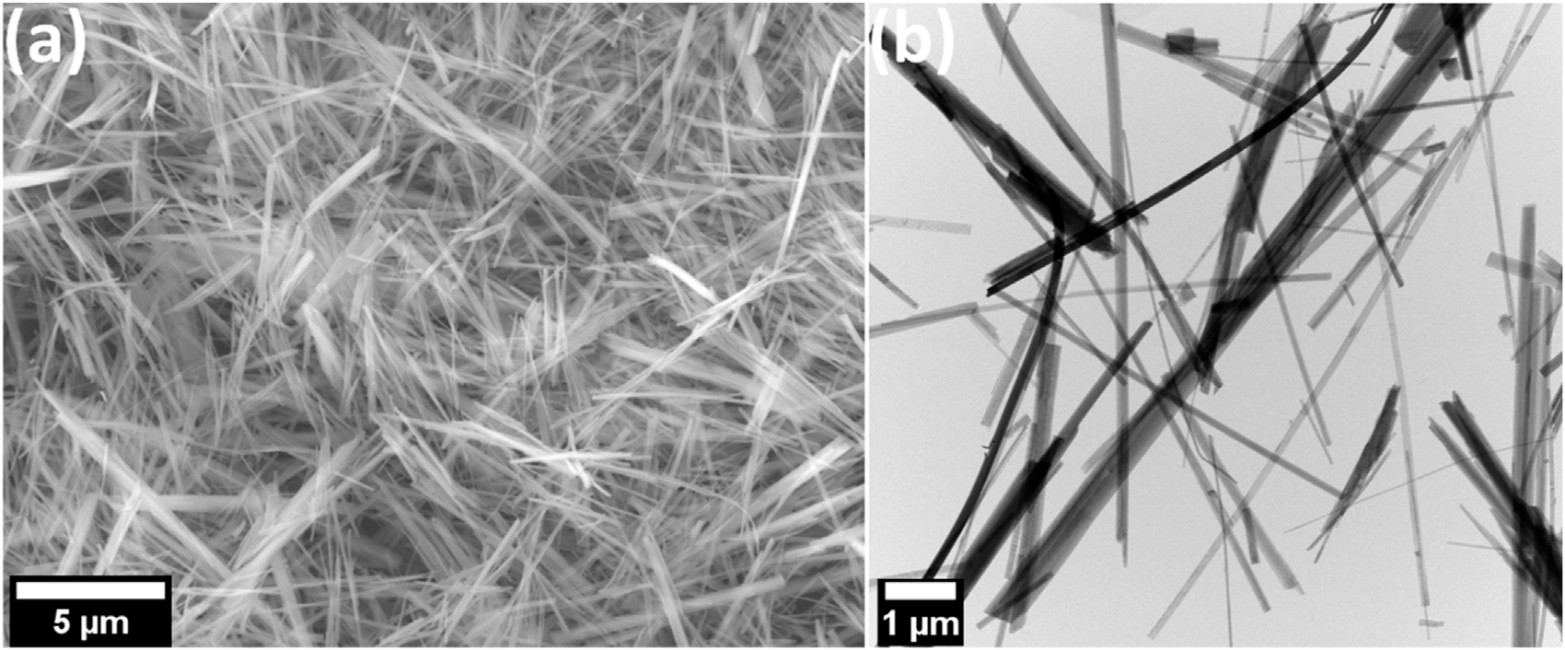
FESEM **(A)** and STEM **(B)** micrographs of HZTO nanowires.

**FIGURE 2:**
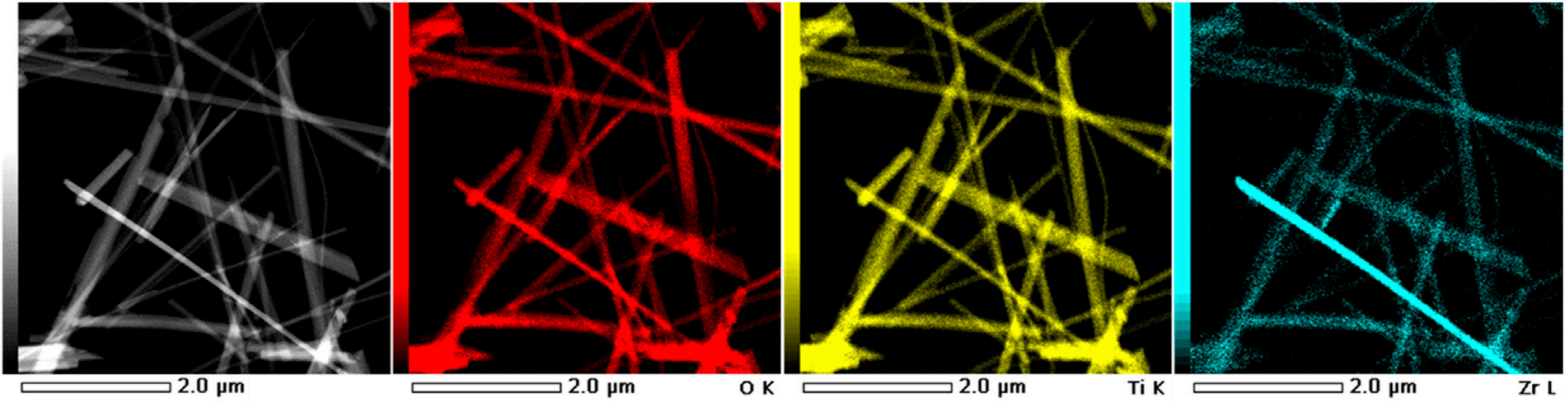
STEM-HAADF image and elemental mapping images of HZTO nanowires. The red, yellow, and blue areas correspond to O, Ti, and Zr elements, respectively.

Figure 1 presents the FESEM and STEM micrographs demonstrate the HZTO nanowires in the free standing form with a high aspect ratio exceeding 50. The comparatively high reaction temperature (240 °C) and/or extended reaction time (48 h) are the causes of this high aspect ratio. Moreover, Figure 2 reveals the STEM-HAADF and elemental mapping images of HZTO nanowires prove the homogeneous distribution of O, Ti, and Zr elements. The detailed structural and morphological properties of HZTO nanowires can be found in Hanani et al. (2022) and Hanani et al. (2021).

### 3.2 Characterization of SA/HZTO beads

**FIGURE 3:**
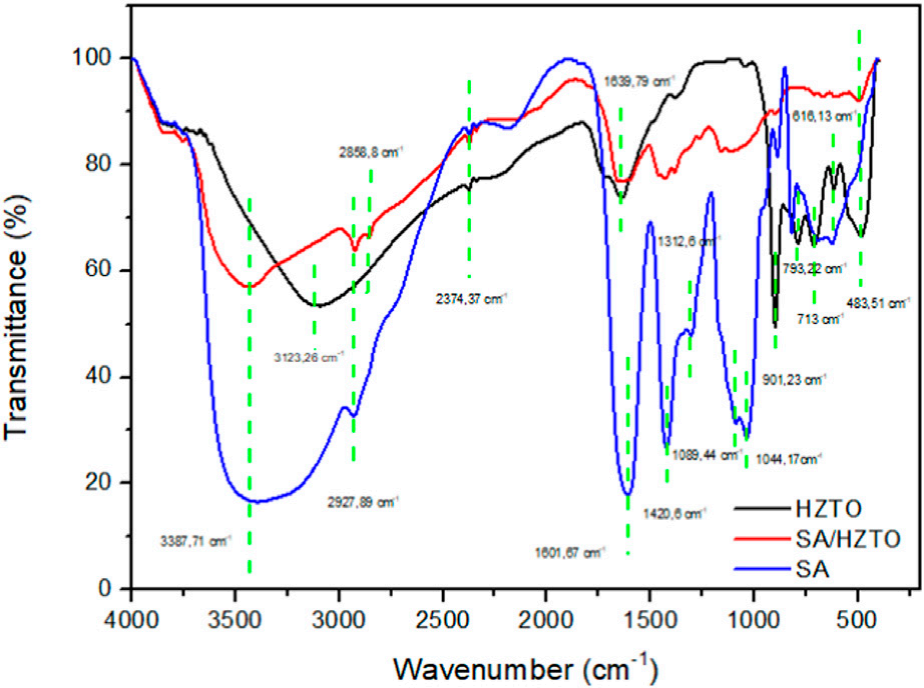
Fourier transform infrared (FTIR) spectra of SA, HZTO nanowires, and SA/HZTO beads.

The morphological structure and functional groups of the SA/HZTO sample were determined by SEM and FTIR analysis, respectively. The SA/HZTO beads were achieved by the interconnecting process of sodium alginate (SA) with Ca^2+^. The final material has white-colored beads after the freeze-drying process. To verify the formation of SA/HZTO beads cross-linked in Ca^2+^ ions, the functional groups of sodium alginate, HZTO nanowires, and SA/HZTO beads were determined by FTIR spectroscopy (Figure 3). The FTIR spectra of calcium alginate are described as following: the broad band around 3387.71 cm^−1^, which corresponds to the stretching vibrations of hydrogen-bonded O-H groups (Fertah et al., 2017). A weak signal at 2927.89 cm^−1^ attributed to the stretching vibration of C-H bonds. An intense band at 1420.6 cm^−1^ and another band at 1601.67 cm^−1^, representing symmetric and asymmetric stretching vibration of the carboxylate group, respectively, indicating specific bonding interactions between COO^−^ and Ca^2+^ (López Córdoba et al., 2013; Ablouh et al., 2019; Bahsis et al., 2020). A smaller peak was observed at 1089.44 cm^−1^, which can be assigned to the stretching of C-O bonds in COH groups, a characteristic feature of the G blocks in calcium alginate. Two peaks with relatively low intensity were observed below 1,000 cm^−1^: the first at 887.73 cm^−1^, indicative of preferential binding involving C-O-C interactions, and the second at 828.17 cm^−1^, associated with the stretching of C-C bonds in G blocks (Nouri et al., 2020).

HZTO infrared spectra illustrate characteristic peaks, of which the peak at 3,123 cm^−1^ corresponds to O-H stretching vibrations (Kong et al., 2020). The weak bands at 616.13 cm^−1^ and 483.51 cm^−1^ correspond to the bending vibration of Ti-O bonds (Nouri et al., 2020).

SA/HZTO bead infrared spectra showing various peaks with the same wavenumber confirm the presence of all the functional groups that are originally present on both HZTO and SA beads. However, variations in intensity were observed.

**FIGURE 4:**
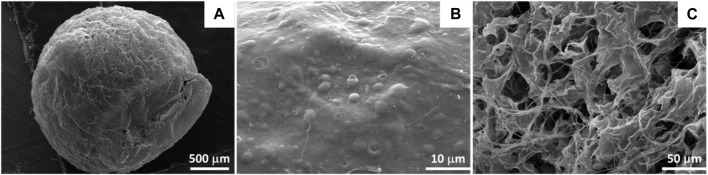
SEM micrographs of the SA/HZTO bead surface **(A,B)** and SA/HZTO bead cross section **(C)**.

Dried SA/HZTO beads (Figure 4A) appear uniform in size and maintain their porous, spherical shapes in scanning electron images. In addition, SEM images (Figure 4B) show the surface morphology of SA/HZTO beads which present a relatively smooth surface and had an orderly homogeneous shape with some creases and apertures. The bead section’s morphological image (Figure 4C) generates a permeable shape that can be attributed to extensive porosity. The pore diameters are quite homogeneous, ranging from 600 nm to more than 50 μm on average. This super porous structure is made up of a loose network of linked fibrils, as a result of the elimination of water into moist beads and the generation of an extensively associated fibril network with the effective freeze-drying process.

**FIGURE 5:**
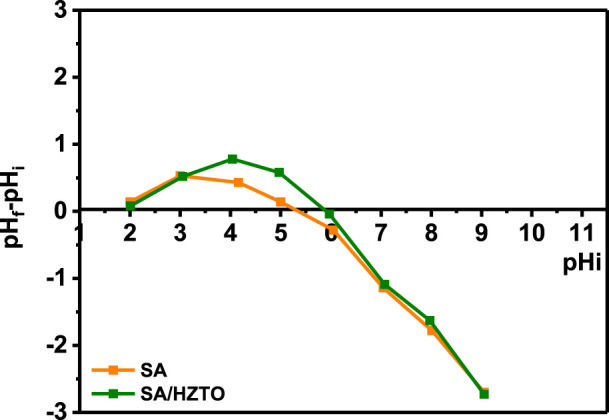
Zero-charge point pH_pzc_ of the SA/HZTO bead adsorbent.

pH_pzc_ determined the surface charge of SA/HZTO beads depending on the pH solution. Figure 5 shows the pH_pzc_ value of SA and SA/HZTO was equal to 5.25 and 5.72, respectively. They are lower than the pH of the adsorbent solution (pH = 6). In addition, when the pH is lower than pHpzc, the adsorbent surface is protonated and negative for a pH higher than pHpzc (when pH_pzc_ < pH, the bead surface was negative, and when pH_pzc_ > pH, the bead surface was positive). In addition, when the pH solution is identical to pH_pzc_, the adsorbent surface group charge is neutral and the electrostatic force is negligible between SA/HZTO and the dye molecules. This makes it possible to say that the functional groups at the surface of these adsorbents (carboxylates) are negatively charged (-COO^-^), which bind electrostatically with positively charged cationic dyes.

### 3.3 Adsorption study

#### 3.3.1 Impact of various parameters on dye removal

**FIGURE 6:**
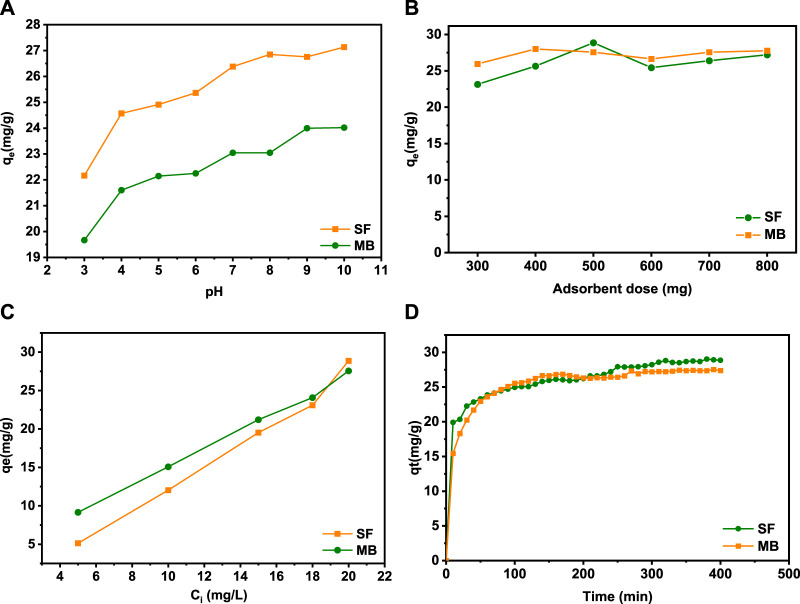
**(A)** Effect of pH, **(B)** amount of adsorbent, **(C)** initial dye concentration, and **(D)** reaction time for the adsorption of MB and SF onto SA/HZTO beads.

**FIGURE 7:**
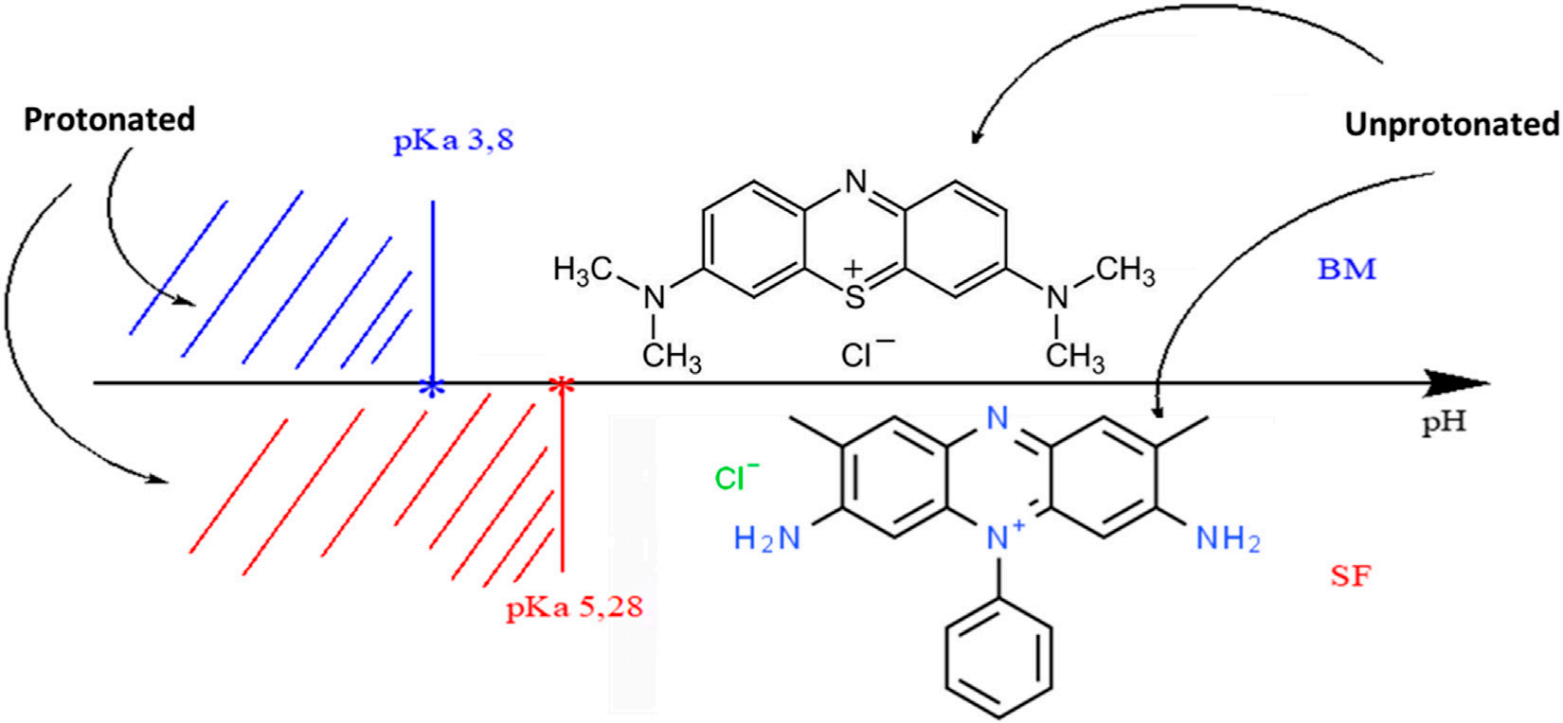
Ionization zone of MB and SF dye molecules.

According to the literature, the pH solution, the amount of the adsorbent, the initial dye concentration, and time contact are the majority critical factors determining the adsorption capacity and removal effectiveness of adsorbent materials. The ionization state of SF and MB in water depends on the pH. To examine the impact of pH on the removal of cationic dyes, tests were realized in the pH range from 3 to 10 (Figure 6A). It can be seen that the elimination of the MB and SF dyes increases progressively with the increase in the pH solution from 3 to 10. The difference in the retention rate at all values of pH is mainly due to the ionization state of each dye. The zero-load point of the SA/HZTO adsorbent was 5.72. In other words, for pH < 5.72, the carboxylate groups will be protonated and beyond that will be in the form of an anion. The adsorbent-adsorbed interactions will take place, essentially, by electrostatic anion/cation interactions. For pH < 5, the dyes and alginate will be protonated, and the interactions will be dipolar and hydrogen bonding in nature (Figure 7). In addition, the effect of the adsorbent dosage on the organic dye removal capacity of the SA/HZTO gel beads was studied in the range of 300 to 800 mg, and the results are shown in Figure 6B. Therefore, the removal capacity of MB and SF was not remarkably changed when the mass of the adsorbent value was above 500 mg. We can say that the adsorbent concentration has a critical threshold, and beyond this threshold, the particles associate (aggregate). The available surface allowing the fixation of the dyes decreases slightly (Figure 6C).

The contact time is one of the primary parameters that impact on the adsorption of organic dyes from an aqueous solution. The adsorption of the two dyes is quick, and a maximum is reached after 30 min (Figure 6D). Beyond that, between 30 and 400 min, the absorption rate is very slow and reaches saturation after 400 min. Therefore, 400 min was fixed as the optimal adsorption time for further experiments.

#### 3.3.2 Adsorption kinetics

To describe the method(s) of retention of the two probes, we have represented the investigational data employing the pseudo-first-order (Eq. 3) and the pseudo-second-order model (Eq. 4) (Tan and Hameed, 2017).

$$\log\left(q_{e}-q_{t}\right)=\log q_{e}-\left(k^{1}t\right)/2.303.$$ (3)

**FIGURE 8:**
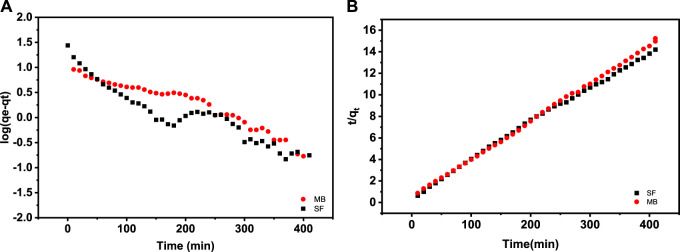
Fitting graphs of adsorption kinetic models: **(A)** pseudo-first-order and **(B)** pseudo-second-order.

Figure 8B shows log (q_e_–q_t_) vs. t plots utilized to determine the parameters of PFO, where k₁ is the pseudo-first-order rate constant derived from the slope of the fitted straight line, and qe, called the adsorption capacity at equilibrium calculated from the corresponding intercept.

The change in slope, for the two dyes, after 200 min of the adsorbent/dye contact can only be explained by a change in the physical state of the adsorbent. As we know, polymers soluble in water start with wetting, followed by swelling, and then the chain dispersion. For the crosslinked SA/HZTO adsorbent, there will be a gradual swelling over time, until a maximum hydrodynamic volume is reached. At this point, the chains in the network begin to relax and will generate a larger contact surface. The change in the slope can only be due to maximum development of the contact surface of the adsorbent. This will allow greater adsorption. This confirms that during the adsorption of the dye on the support, there is intervention of the two dye/support partners in the kinetics of adsorption.

$$t/q_{t}=\frac{1}{k^{22}q_{e}}+\frac{t}{q_{e}}.$$ (4)

The parameters of the PSO model resulted from the intercept and the slope of the fitted straight line of (t/q_t_) versus t shown in Figure 8A, where k₂ is the PSO rate constant. Table 1 lists the parameters of MB and SF adsorption kinetic models. As seen, the correlation coefficients (R^2^) obtained from the PSO model for MB and SF adsorption equaled 0.999 and 0.997, respectively. The discrepancy between q_e_ from the PSO model and q_e_ experimental values is less than that of the pseudo-first-order model. Therefore, the PSO model well-described the experimental investigation. Similar observations have been suggested to describe the adsorption kinetics of both SA and MB organic dyes (Trinh et al., 2021), (Minisy et al., 2021). This result confirms what we have advanced on the SF and MB ionization state.

**TABLE 1:** Kinetics parameters for SF and MB adsorption onto the SA/HZTO adsorbent.

Model		Parameter	MB	SF
Pseudo-first-order	Linear fit 1 (0–190 min)	q_e_ (mg/g)	6.93	13.42
		k₁ (min⁻^1^)	4.88 10⁻³	16.85 10⁻³
		R^2^	0.95	0.99
	Linear fit 2 (260–410 min)	q_e_ (mg/g)	51.70	16.39
		k₁ (min⁻^1^)	13.97 10⁻³	11.79 10⁻³
		R^2^	0.95	0.80
Pseudo-second-order		q_e_ (mg/g)	28.28	30.03
		k₂ (g mg⁻^1^min⁻^1^)	0.278	0.219
		R^2^	0.99	0.99

#### 3.3.3 Thermodynamic study

To gain insights about the effect of the temperature on the SF and MB adsorption process, several tests were executed at 303, 313, 223, and 333 K. The Ln (Kc) vs. 1/T plots were used to obtain ΔH° and ΔS°. The enthalpy was determined from the slope and the entropy derived from the corresponding intercept. ΔG° is determined from the equilibrium constant Kc using Eqs 5, 6. The standard values of entropy (ΔS°), enthalpy (ΔH°), and free energy (ΔG°) at different temperatures are given in Table 2:

$$Kc=\frac{q_{e}}{C_{e}},$$ (5)

ΔG°=−RTlnKc. (6)

**TABLE 2:** Thermodynamic parameters of MB and SF elimination onto SA/HZTO.

Dye	Temperature (K)	ΔG° (kJ/mol)	ΔH° (kJ/mol)	ΔS° (J/mol K)
SF	333	7.400	−110.74	−348.27
	323	−0.671		
	313	−2.810		
	303	−3.380		
MB	333	0.831	−40.03	−123.63
	323	0.242		
	313	−1.118		
	303	−2.771		

The enthalpy (ΔH°) value is indicating the exothermic nature of the MB and SF removal onto SA/HZTO gel beads and a physical adsorption mechanism (Maaloul et al., 2021). The negative ΔS° supports the fact that adsorption increases the overall order of the system and is not in favor of a spontaneous process. However, the energy balance of the system is exothermic and is favorable to the adsorption of the two dyes. Indeed, the negative value of ΔG° below the temperatures 333 and 323 K for SF and MB, respectively, indicates that the adsorption is spontaneous.

#### 3.3.4 Adsorption capacity of SA/HZTO for the binary SF/MB system

Despite the fact that SA/HZTO has been demonstrated to be efficient for the removal of one dye, there is a possibility it will face some defiance when subjected to an aqueous solution including two dyes. To treat this issue, SF and MB were jumbled to produce a mixed dye solution. Through this study, we want to know which of the dyes will be more adsorbed than the other if they are both in the presence of the adsorbent. To do this, various combinations and ratios of the two dyes. Table 3 shows the adsorption capacity values at different times in a ternary system (SA/HZTO/MB/SF). The two pigments, taken individually, show similarities in the evolution of the adsorbed amounts over time. These tests were made at room temperature and pH 6, where the two dyes will be positively ionized and the support will be negatively ionized. However, theoretically, the two probes, having different structures, will not have the same affinity with respect to the support because of their different steric hindrance. This explains the slightly different adsorption patterns of the two dyes.

**TABLE 3:** Adsorption capacity at different times of MB and SF on SA/HZTO beads in the binary system.

Binary system		q_t_ (mg/g)			
		10 min	120 min	300 min	400 min
MB(20 mg/L) + SF(5 mg/L)	MB	34.80	39.06	39.99	39.92
	SF	11.67	12.96	13.22	13.12
MB(20 mg/L) + SF(10 mg/L)	MB	35.15	39.37	40.26	40.48
	SF	19.00	21.45	21.95	22.21
MB(10 mg/L) + SF(20 mg/L)	MB	20.65	21.90	22.55	22.42
	SF	32.91	37.43	38.50	38.96
MB(5 mg/L) + SF(20 mg/L)	MB	13.00	13.75	13.92	13.93
	SF	33.24	37.91	38.75	38.38
MB(20 mg/L) + SF(20 mg/L)	MB	34.52	34.96	39.86	39.76
	SF	31.94	37.48	38.25	38.41

If we mix the two dyes at equal concentrations, 50% MB/50% SF, we obtain the same value of the adsorbed quantity. We can say that the two dyes are adsorbed at the same rate, and therefore, they have the same affinity for the support. In this case, the adsorption capacity values of MB and SF are 39.76 and 38.41 mg/g, respectively.

### 3.4 Comparison study

Numerous study researchers have investigated a range of adsorbent materials for the removal of MB and SF from aqueous solutions. Table 4 provides a comparative overview of the maximum adsorption capacity of MB and SF onto various adsorbents.

**TABLE 4:** Comparative overview of the maximum adsorption capacity of MB and SF onto various adsorbents.

Adsorbent	qe (mg/g)		References
	MB	SF	
Compost biochar	13	—	Kujawska and Wasag (2021)
Natural zeolite	19.94	—	Han et al. (2009)
Kaolinite clay	—	16.23	Adebowale et al. (2014)
Alkali-treated mango seed integuments	—	23.3	Malekbala et al. (2012)
Sugarcane bagasse	13.4	—	Meili et al. (2019)
Soursop residues	13.9	—	Meili et al. (2019)
Carbon nanotubes	35	—	Yao et al. (2010)
Polypyrrole nanofiber/Zn-Fe-layered double hydroxide nanocomposite	—	13.76	Mohamed et al. (2018)
Sodium alginate/titanium dioxide nanowire doped with zirconium aerogel beads	40.48	—	This study
Sodium alginate/titanium dioxide nanowire doped with zirconium cryogel beads	—	38.96	This study

### 3.5 Reusability

**FIGURE 9:**
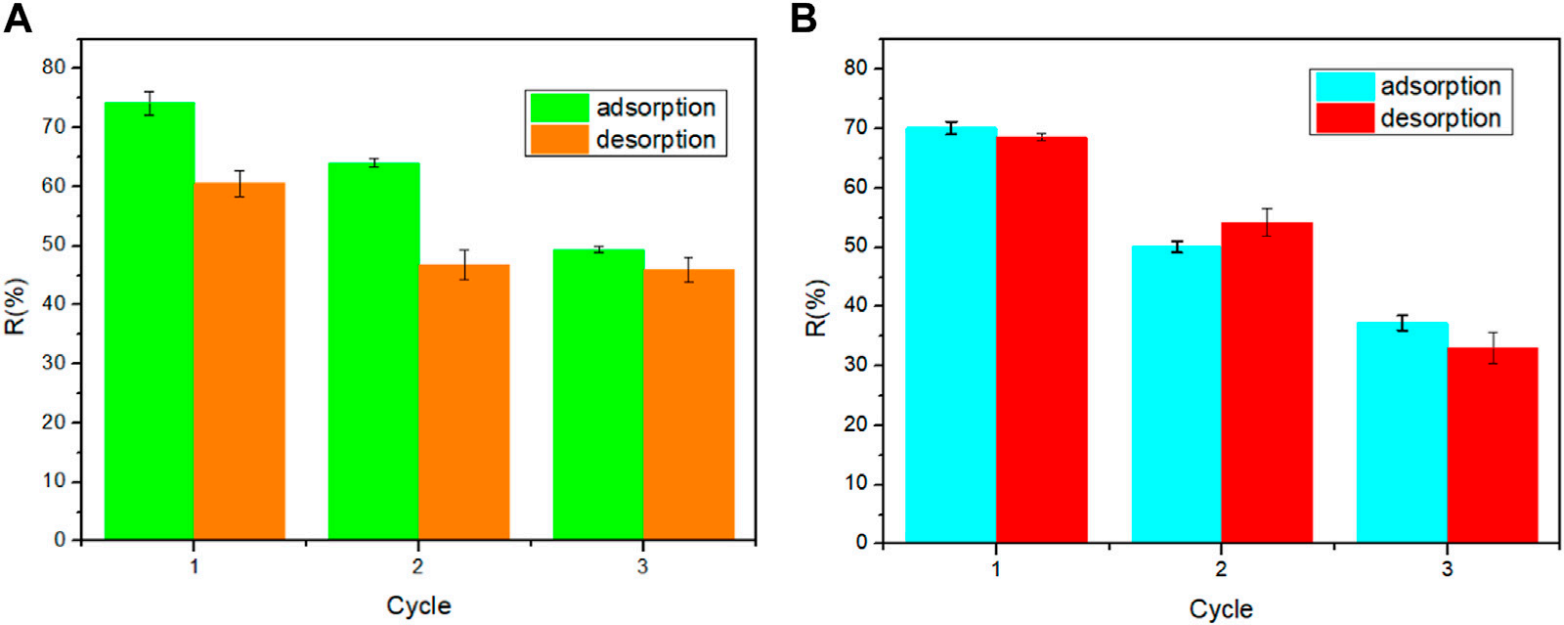
Adsorption–desorption cycles of **(A)** SF and **(B)** MB.

SA/HZTO was regenerated with the application of HCl because the primary interactions between the two organic dyes and adsorbent are specifically cation–anion interactions; it would be introducing a competing cation that can displace the cations already bound to the adsorbent (Azeroual et al., 2023). After the regeneration cycles, SF and MB dyes for SA/HZTO were reduced from 74.18% to 50% and 70.12% to 37.24%, respectively, as shown in Figures 9A, B.

Figure 9 demonstrates that SA/HZTO displays a reversible adsorption phenomenon. This regenerative cycle suggests that SA/HZTO adsorbents can be reused and restored for the uptake of dyes. Hence, the SA/HZTO beads show potential to be a promising and reusable adsorbent for the treatment of wastewater.

## 4 Conclusion

To facilitate separation and improve their adsorption capacity to remove organic dyes (safranin and methylene blue) from an aqueous solution in both single and mixed systems, this study uses green and cost-effective support gel beads made of sodium alginate and titanium dioxide doped with zirconium and cross-linked with calcium chloride. The experimental factors including pH, adsorbent amount, initial dye concentration, and contact time were determined precisely. At basic pH values, the removal of the organic dyes is carried out electrostatically. Thermodynamic calculations indicate an exothermic and spontaneous adsorption process for the two cationic dyes. The kinetic data appropriate well with the PSO adsorption kinetic model. The adsorption capacities, in the presence of both SF/MB dyes, indicate that there is no support preference for either of the two dyes and that the evolution of the adsorption has the same rate and depends only on the concentration. In summary, SA/HZTO with a large adsorption capacity may be a favorable adsorbent for the efficient elimination of single or binary organic dyes. In addition to the selective recuperation of a cationic colorant from water, the catalytic and photocatalytic activity of this absorber can be researched.

## Data Availability

The original contributions presented in the study are included in the article/Supplementary Material; further inquiries can be directed to the corresponding authors.
